# Development of Multiple Nucleotide Polymorphism Molecular Markers for Enoki Mushroom (*Flammulina filiformis*) Cultivars Identification

**DOI:** 10.3390/jof9030330

**Published:** 2023-03-07

**Authors:** Fei Liu, Shi-Hui Wang, Ding-Hong Jia, Hao Tan, Bo Wang, Rui-Lin Zhao

**Affiliations:** 1 State Key Laboratory of Mycology, Institute of Microbiology, Chinese Academy of Sciences, Beijing 100101, China; 2 School of Plant Protection, Jilin Agricultural University, Changchun 130118, China; 3 National-Local Joint Engineering Laboratory of Breeding and Cultivation of Edible and Medicinal Fungi, Sichuan Institute of Edible Fungi, Sichuan Academy of Agricultural Sciences, Chengdu 610066, China; 4 College of Life Science, University of Chinese Academy of Sciences, Beijing 100049, China

**Keywords:** mushroom, MNP marker, sequencing, cultivar

## Abstract

The enoki mushroom (*Flammulina filiformis*) is one of the most important and popular edible mushrooms commercially in China. However, traditional mushroom cultivar identification is challenging due to poor accuracy, heavy workloads, and low reproducibility. To overcome this challenge, we developed a method for identifying *F. filiformis* strains using multiple nucleotide polymorphism sequencing (MNP-seq). This involved screening 179 universal MNP markers based on whole-genome sequencing data, constructing an MNP sequence library, and performing multiplex PCR amplification and high-sequencing. We further screened 69 core MNP markers and used them to build a neighbor-joining (NJ) phylogenetic tree of 232 cultivated and wild strains. Our analysis showed that all cultivars could be accurately separated by computing genetic similarity values and that the cultivars could be separated into 22 distinct evolutionary pedigrees. The specific value of genetic similarity can be used as the standard to distinguish *F. filiformis* cultivars, however, it needs to be comprehensively defined by the additional phenotype and biological characteristics of those strains in the future work.

## 1. Introduction

*Flammulina* is a genus of edible mushrooms that belongs to the phylum Basidiomycota and the family Physalacriaceae. There are about 20 *Flammulina* species that have been described [1]. Some of the well-known species of *Flammulina* include *F. filiformis*, *F. populicola*, *F. hispida*, and *F. tabacina*. *Flammulina filiformis* (Z.W. Ge, X.B. Liu & Zhu L. Yang) P.M. Wang, Y.C. Dai, E. Horak & Zhu L. Yang, also known as enoki mushrooms in western and winter mushrooms or golden needling mushrooms in China, is one of the most important and popular edible mushrooms available commercially [2]. It is widely cultivated and consumed because of its nourishing qualities and desirable taste [3]. Enoki mushrooms grow naturally on Chinese hackberry tree stumps. There is a clear morphological difference between naturally grown and domesticated strains. Wild enoki mushrooms have yellowish to brown basidiocarps, while cultivated strains have white, thin, and slender stems [4]. Enoki mushrooms were first cultivated in China during the eighth century and then spread to Japan. It has been cultivated on wood logs under semi-wild conditions for over 300 years. The use of bottle cultivation technology in enoki cultivation has become increasingly popular in recent years. Up until the 1990s, Japan dominated the world’s enoki mushroom production. Since then, China has replaced Japan as the world’s largest producer. Currently, most of China’s enoki production units are fully mechanized, with an annual production capacity of 2.4 million tons [3]. Previously, *F. filiformis* from eastern Asia was named as *F. velutipes* (Curtis) Singer, which was a species that originated in Europe [5,6]. Recently, phylogenetic results revealed that “*F. velutipes*” in eastern Asia is not identical to the European *F. velutipes* and should be treated as a separate species, namely *F. filiformis* [7,8].

Mushroom researchers and cultivators commonly bred mushroom strains by tissue isolation and developed the new cultivars by the systematic selection method, which makes strains that are genetically very homogeneous. On the other hand, traditional mushroom cultivar identification is challenging due to poor accuracy, heavy workloads, and low reproducibility [9]. Inconsistent nomenclature of *F. filiformis* cultivars in circulation has led to much confusion in the cultivar names [7,8]. The mushroom industry is one of the many industries facing challenges in protecting patents for commercial cultivars, as it is generally difficult to do so in many countries [10]. One of the main reasons for this difficulty is the challenge of morphologically distinguishing between different cultivars, including original, newly bred, or essentially derived cultivars from previously patented ones, which are required to demonstrate novelty and non-obviousness for patentability. This can result in the unauthorized propagation and sale of cultivars, disputes over intellectual property rights, and a lack of incentives for breeders to develop new cultivars. Therefore, accurately and efficiently determining cultivars is essential for cultivating and breeding *F. filiformis* strains.

Previously, researchers have performed many studies on the identification of *F. filiformis* strains with different genetic markers, such as Restriction Fragment Length Polymorphism of PCR products (PCR-RFLP) [11], Inter Simple Sequence Repeats (ISSR) [12], and Sequence Characterized Amplified Region (SCAR) [13]. ITS-PCR-RFLP offers the advantage of being simpler, cheaper, and especially useful for the routine analysis of large numbers of strains [14]. PCR-RFLP analysis of the ITS regions succeeded in recognizing differences between commercial cultivars and wild-type strains [11]. The genetic diversity of 59 strains was analyzed by the use of ISSR markers and morphological characteristics [15]. However, weak polymorphism, laborious and unstable reproducibility may limit these marker technologies [16]. The most accurate but laborious method to identify differences at the molecular level is directly sequencing cloned genes or PCR products. At present, the multiple nucleotide polymorphism (MNP) marker method has recently been developed and successfully applied to the variety identification of plants [17] and an edible fungus, *Lentinula edodes* [9]. The Chinese national technical standard for plant identity determination now uses the MNP-Seq method, which is based on the presence of multiple SNPs in the genome. By analyzing a combination of unique alleles with distinct SNPs, this method can effectively differentiate between different individuals. The efficiency of MNP-Seq was attributed to multiplex PCR, high-throughput sequencing, and bioinformatics analysis. The multiplex PCR in the first step had a high efficiency to enrich thousands of marker loci by a single PCR reaction [18]. High-throughput sequencing is a technology that allows for the rapid and simultaneous sequencing of large amounts of DNA, and it has revolutionized genomics research. Bioinformatics, on the other hand, is a field that combines computer science, statistics, and biology to analyze and interpret biological data. It is used to analyze the data generated by multiplex PCR sequencing and to identify the specific genetic markers that are unique to each cultivar. The advantage of the MNP method is that it is more accurate and accessible than other molecular marker methods, such as Simple Sequence Repeats (SSR) markers, which were previously used for identifying *F. filiformis* [7,19]. The phenotypic and genetic diversity of 37 *F. velutipes* strains were investigated using seven agronomic traits and 70 SSR markers, respectively, to find elite breeding strains of *F. velutipes* strains [7]. A total of 12 polymorphic SSR markers were developed from an SSR-enriched library of *F. velutipes* SSR, and these markers were used to analyze the genetic diversity of 32 strains of *F. velutipes* from Korea, China, and Japan [20]. To understand the genetic background and breeding history of *F. velutipes*, 124 cultivars and wild strains were tested, and 25 SSR polymorphic markers were developed [21]. The genome sequencing of *F. filiformis* [22,23,24] aids in the development of large numbers of SSRs to identify *F. filiformis* strains. However, repetitive SSRs can induce DNA polymerase slippage during polymerase chain reaction, introducing erroneous SSR alleles to analysis [25,26].

In this study, we utilized the MNP marker method to investigate the mushroom-forming fungus *F. filiformis*. To achieve this, we generated 179 MNP markers based on 232 genomic sequences of this species and subsequently identified 69 core MNP marker sequences that were utilized for phylogenetic analysis to reveal their evolutionary relationships. Additionally, we devised a streamlined approach for identifying *F. filiformis* cultivars by computing genetic similarities between different cultivars and lineages. It is important to note that although we only utilized the MNP-Seq method for *F. filiformis* in this study, the tool has broader applications and can also be used to analyze other mushroom species.

## 2. Materials and Methods

### 2.1. F. filiformis Strains, DNA Extraction, and Whole-Genome Sequencing

*F. filiformis* strains collected in the study are shown in Table 1 and Table 2. In total, 232 strains of *F. filiformis* strains were collected in this study, including 157 cultivars and 75 wild strains. To obtain a comprehensive understanding of the genetic variation present within the *F. filiformis*, we selected cultivars from various countries, although a significant proportion of the strains were sourced from China. The mycelia were grown in solid Potato dextrose agar (PDA) medium at 25 °C until they reached full growth. Genomic DNA was extracted from mycelia using the CTAB method [27]. A Nanodrop and 1.0% agarose gel electrophoresis were used to assess the concentration and integrity of the DNA solution. Whole-genome sequencing libraries were prepared using NexteraXT reagents (Illumina). The Illumina Novoseq platform from Novogene was then used for sequencing the DNA samples. Briefly, approximately 2 μg of DNA from each sample was used for fragmentation by Biorupter (high power: (15 s, on/90 s, off), six cycles) and end preparation by NEXT flex TM End-Repair. After PCR amplification (10 cycles), the library was purified using AMPure beads. Qubit was used to evaluate the quality and quantity of each library. The sequencing statistics of the samples are summarized in Table 1.

### 2.2. Screening and Primer Design for MNP Markers in F. filiformis

Sequence artifacts, including reads containing adapter contamination, low-quality nucleotides, and unrecognizable nucleotide (N), undoubtedly set the barrier for the subsequent reliable bioinformatics analysis. Hence, quality control is an essential step to guarantee meaningful downstream analysis. Fastp (version 0.19.7) [28] was used to perform basic statistics on the quality of the raw reads. The steps of data processing were as follows: (1) Discard a paired-read if either one read contains adapter contamination; (2) Discard a paired-read if more than 10% of bases are uncertain in either one read; (3) Discard a paired-read if the proportion of low quality (Phred quality < 5) bases is over 50% in either one read. In total, 163 whole-genome resequencing data of *F. filiformis* were analyzed. The sequencing data were mapped to the *F. filiformis* reference genome (accession number: AQHU01) with BWA (version 0.7.17-r1188) [29] with the parameters ‘‘bwa mem -t 8 -R”. SNPs were then identified with samtools [30]. A sliding window of 130 base pairs was used to scan all SNP-containing genome segments with an increment of 10 bp. The discriminative power (DP) of a window was defined as t/c(N,2), where c(N,2) was the number of variety pairs among N varieties used and t was the number of the teams, each of which had at least two dispersed SNPs within the window. The windows with DP > 0.4 were chosen for multiplex PCR primer design and synthesis at BGI Genomics.

### 2.3. Library Construction and MNP Sequencing

All primers were diluted to 100 μM, and then 5 μL of each primer was pipetted into the primer mix pool. The multi-PCR reaction system consisted of 12 μL Template DNA, 5 μL Primer Mix, 5 μL 10 × Multi HotStart Buffer, 4 μL Super Pure dNTPs, 1 μL Multi HotStart DNA Polymerase, and 27 μL ddH_2_O. The total volume of each reaction mixture was 50 μL. The PCR reactions were performed as follows: 95 °C for 15 min; followed by 25 cycles at 94 °C for 30 s 58 °C for 90 s, and 72 °C for 60 s; followed by elongation at 72 °C for 10 min; and finally cooling to 4 °C. After the reaction, the PCR products were purified using the paramagnetic particle method. A total amount of 1.5 μg DNA per sample was used as input material for library construction. Sequencing libraries were generated using NEBNext Ultra DNA Library Prep Kit for Illumina (NEB in Ipswich, MA, USA, E7370L) following the manufacturer’s recommendations, and indexes were added to attribute sequences to each sample. Briefly, the DNA samples were end-polished, A-tailed, and ligated with the full-length adapter for Illumina sequencing. Subsequently, the DNA products were purified by AMPure XP system (Beckman Coulter Life Sciences in Beverly, MA, USA), and size distribution was analyzed by Agilent 5400 system (Agilent Technologies in Santa Clara, CA, USA) and quantified by qPCR (1.5 nM). Qualified libraries were mixed at equal mass (100 ng) and sequenced by the Illumina Novoseq platform from Novogene. The sequencing data volume for each strain was set at 1000 M.

### 2.4. Core MNP Markers and Pedigree Determination

We chose the core MNP markers from all MNP markers with a 100% amplification rate for all strains tested. With the amplified sequences of these core markers, a phylogenetic tree was constructed using the NJ method and ITOL [31], which further differentiates the pedigree of all commercial cultivars.

### 2.5. Genetic Similarity (GS) Calculation

We mapped each sample’s multiplex PCR sequencing and whole genome sequencing results to the reference genome Fv6-3 (AQHU01) with BWA (version 0.7.17-r1188) [29] with the parameters ‘‘bwa mem -t 8 -R” and the consensus sequence was obtained. All MNP sequences from all the samples were extracted based on the location information of the core MNP markers. We evaluated pairwise comparisons of the core MNP sequences from all samples. Each of the paired samples with identical sequences at the same MNP locus was supposed to have the same genotype. We calculated the genetic similarity (*GS*) between two strains according to the formula: the number of identical MNP sequences between two strains divided by the number of core MNP sequences.

## 3. Results

### 3.1. Genome Resequencing, Screening of Universal MNP Markers, and MNP-Seq

We sequenced 163 *F. filiformis* strains and obtained about 5 Gb of clean data per sample (Table 1). We assessed the quality of the sequencing data by mapping reads to the *F. filiformis* reference genome Fv6-3 (NCBI accession no. AQHU01). The mean genome coverage was 90.5%, the mean depth was 133.0, and the average mapping rate of reads was 85.8%. Since the data was sufficient, we used this sequencing data to screen for MNP markers.

First, we selected 179 universal MNP markers based on genomic screening (see Method). Then, we designed and synthesized primers and performed multiplex PCR amplification and sequencing for 69 strains. The mean clean data per strain was 1.3 Gb, the mean Q20 value was 97.7%, and the mean Q30 value was 93.1% (Table S1). In sum, 9583 markers were detected, with an average sequencing coverage of 21,951-fold per strain (Table 2).

### 3.2. MNP Markers Evaluation

**Figure 1:**
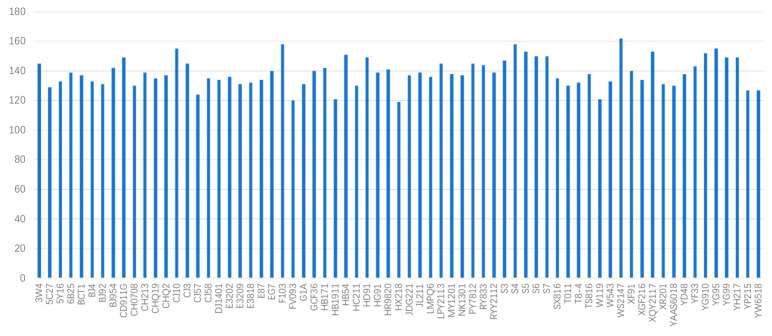
Distribution of the number of detected MNP markers in *Flammulina filiformis* strains.

MNP markers were detected in a range of 119 to 162, with an average of 138.9 markers per strain. The distribution of MNP markers detected in each strain is presented in Table 2 and Figure 1. To verify the consistency of the MNP-seq data, twenty-five strains were randomly selected for both MNP-seq and whole genome sequencing, and the MNP markers from both data sets were compared. The comparison revealed that all the MNP markers detected by MNP-seq in each strain were covered by the whole genome sequencing data, indicating a 100% reproducibility rate.

### 3.3. Construction of Phylogenetic Relationship Using Core MNP Sequences

**Figure 2:**
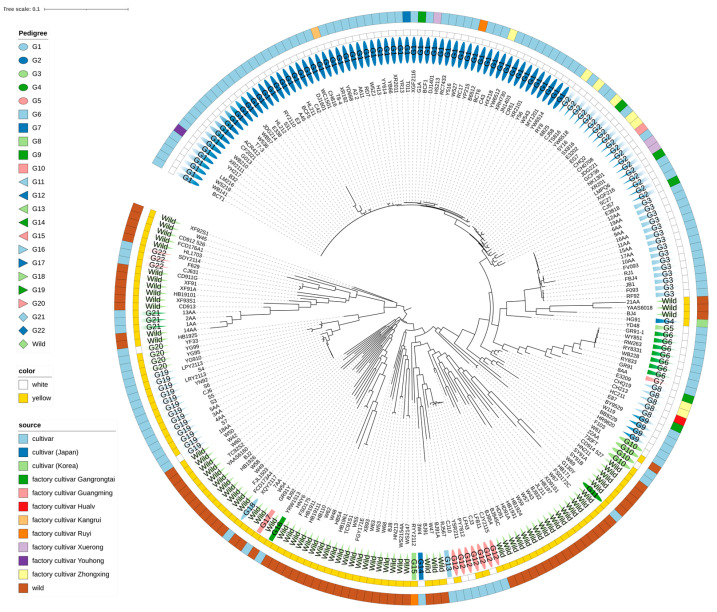
Phylogenetic tree of *F. filiformis* based on 69 core MNP sequences from 232 *F. filiformis* strains. For each strain, the innermost color ring represents the pedigree of the strain, the second ring indicates pileus color, and the outer ring indicates the original source of the strain.

We successfully detected 69 MNP markers in all strains from 179 universal MNP markers, and these markers were chosen as core MNP markers. We constructed an NJ phylogenetic tree using these core MNP sequences from 232 *F. filiformis* strains (Figure 2), including 69 MNP-seq data and 163 whole genome sequencing data. All cultivars could be recognized as one of 22 lineages (Figure 2).

### 3.4. Genetic Similarity Values between Different Cultivars and Lineages

**Figure 3:**
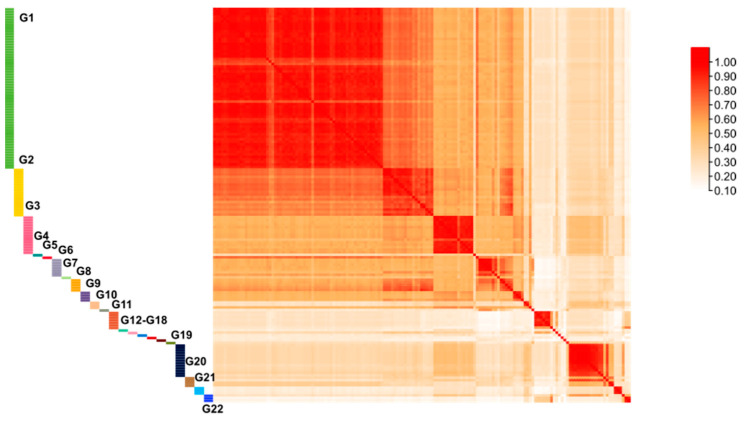
The heatmap of the *GS* values of pairwise comparison between 22 pedigrees and all cultivars.

The *GS* values were computed between each pair of cultivars and all pairwise lineages. There are potentially 22 different pedigrees that can be used to distinguish all *F. filiformis* cultivars (named G1-G22, respectively, in Figure 3 and Table S2). Within the same pedigrees, the *GS* values for various cultivars were all more than 60%. Pedigrees 1, 2, and 19 showed the highest (mean *GS* > 91%), while Pedigrees 10, 20, and 22 had the lowest genetic diversity values (mean *GS* < 72%). The minimum genetic similarity values between pedigrees (Table S3) and cultivars showed that these pedigrees could be distinguished by a *GS* value of less than or equal to 60%, and the *GS* value between strains with the range of 60–98.6%, they could be identified as different cultivars but in the same pedigree. 

## 4. Discussion

*Flammulina filiformis* is one of the most widely cultivated mushrooms in the world on a large commercial scale. It was reported that the first cultivar in China was domesticated from a wild strain isolated from Fujian Province in 1974 [32]. In 1983, Fujian breeders introduced the first white strain from Japan [21,32]. Four years later, in 1987, F21, another strain with white and slender stem characters, was introduced in China. This pattern indicates that the white strains in China were probably originally introduced from Japan, and the yellow strains may have been domesticated directly from the wild strains [21,33].

In this study, we found that most white cultivars were from Pedigree 1, Pedigree 2, and Pedigree 3, and they have close evolutionary relationships, especially for Pedigree 1 and Pedigree 2. In contrast, the yellow cultivars were often clustered with wild strains, which is consistent with previous studies that the yellow cultivars were directly domesticated from wild strains isolated from China or hybridized between white and yellow strains [21]. For example, the yellow cultivar G130Y from Pedigree 11 was clustered with the wild strain HB171, cultivar YRW1513 from Pedigree 16 was clustered with the wild strain HNY6, and cultivars SDY2114, F629, and CJ631 from Pedigree 22 were clustered with the wild strain HL1703. Generally, the white cultivars and the wild strains are separated clearly in our phylogenetic tree using core MNP markers. The high genetic diversity in wild populations (Figure 2) suggests that a large gene pool in nature is available for mushroom breeding, which is consistent with the previous study of *F. filiformis* using SSR markers in China [21]. Additionally, strains from the same region or country were assigned to different pedigrees, indicating that the genetic distances are not correlated with geographic origins.

Interestingly, we found that many white cultivars grown in different factories belong to the same pedigree. For example, strains YH217 from Youhong, T8-4 from Kangrui, and DJ1401 from Xuerong are from Pedigree 1; strains TS816 from Zhongxing and E3202 from Gangrongtai are from Pedigree 2. White cultivars grown in different factories sharing the same ancestry might indicate that they were originally introduced from the same strain [20]. It is also possible because they have been intentionally bred to have similar traits, such as color, disease resistance, or yield potential. In this case, breeders might use the same parent or closely related strains to develop different cultivars with similar traits. In addition, *GS* values of 100% for some cultivars in this study indicate that they share the same genetic origin, but they were given distinct names: cultivars XR2111 and WB210 from Pedigree 1 are the same; cultivars 531 and 6B25 from Pedigree 2 are the same. Further cultivation experiments will be required to determine whether they are the same cultivars.

The efficiency of MNP-Seq was attributed to multiplex PCR, high-throughput sequencing, and bioinformatics analysis. A single PCR reaction enriched thousands of marker loci by multiplex amplification in the first step [17]. Combining deep sequencing and bioinformatics analysis with MNP-Seq software, we could genotype more than 1000 MNP markers for *F. filiformis* in only one day. In our experience, MNP-seq has many advantages over other methods. It requires less starting DNA to amplify the MNP markers using mixed MNP marker primers. The high-throughput sequencing-based detection of MNP markers overcomes the uncertainty of SSR amplification length displayed on gel electrophoresis. MNP markers are often sequenced thousands of times, improving reproducibility and accuracy. Compared with whole-genome sequencing-based SNP markers, MNP-seq requires less experimental and data analysis time. Due to the high reproducibility and accuracy of MNP-seq, no replicate was required for MNP genotype determination [17].

## 5. Conclusions

The identification of different strains of enoki mushroom by MNP molecular markers is a systematic work. In this study, we have established the relevant MNP sequences library by using 69 pairs of primers, built the phylogenetic trees, and calculated the pair genetic similarity values of all strains. The results showed that most strains could be distinguished well by the phylogenetic topology and different genetic similarity values. The specific value of genetic similarity can be used as the standard to distinguish *F. filiformis* cultivars, however, it needs to be comprehensively defined by the additional phenotype and biological characteristics of those strains in the future work.

The development of MNP molecular markers is a promising approach for accurately identifying enoki mushrooms. MNP markers are based on variations in the DNA sequence, which can be used to distinguish different strains of mushrooms. The use of MNP markers has several advantages over traditional methods, including high accuracy, reproducibility, and ease of use once the identification system was established. However, there are some limitations to this approach. One limitation is the need for specialized equipment and bioinformatic expertise to develop those MNP markers. Another limitation is the availability of reference sequences for different strains of enoki mushrooms. More research is needed to expand the database of reference sequences and to develop a standardized protocol for MNP marker analysis. Future studies should focus on optimizing the MNP-seq method for enoki mushroom identification and developing a user-friendly tool for mushroom growers and researchers. This would enable accurate and rapid identification of different strains of enoki mushrooms, which could have important implications for their breeding, cultivation, and commercialization. Additionally, further investigation into the genetic diversity of enoki mushrooms would help to better understand the molecular basis of this species and its potential for future breeding programs.

## Figures and Tables

**Table 1:** The basic information of whole genome sequenced strains.

Sample	Clean Base (bp)	Average Mapping Rate of Reads	Mean Genome Coverage	Mean Depth	Cap Color	Source	Location
W67	4,925,351,700	80.00%	94.00%	221.7	yellow	wild	Beijing
21AA	6,042,946,800	91.90%	88.80%	193.3	yellow	wild	Beijing
SY91B	6,063,577,800	91.10%	88.90%	168.3	yellow	wild	Liaoning
HD91A	5,816,764,800	89.10%	88.90%	165.8	yellow	wild	Sichuan
XF92S1	5,529,307,200	91.80%	88.80%	165.6	yellow	wild	Xinjiang
XF93S1	4,779,288,600	90.90%	88.80%	159.5	yellow	wild	Xinjiang
W63	5,173,407,000	90.00%	88.70%	158.5	yellow	wild	Hebei
FCD173A4	5,787,528,300	91.30%	88.20%	157.8	yellow	wild	Chengdu
W57	4,929,271,800	91.40%	88.80%	157.6	yellow	wild	Hebei
W58	4,854,398,100	90.90%	88.70%	156.5	yellow	wild	Hebei
YAAS6160	5,502,118,200	89.80%	88.70%	156.3	yellow	wild	Yunnan
HN211	7,548,748,200	90.90%	88.70%	154.7	yellow	wild	-
TC91S1	5,705,983,200	91.60%	89.20%	154.7	yellow	wild	Sichuan
14AA	6,023,080,200	92.70%	88.70%	152.7	yellow	wild	Beijing
BJ8	6,255,115,800	87.20%	91.00%	151.8	yellow	wild	Beijing
WS2154A	6,028,663,200	91.90%	88.70%	149.5	yellow	wild	Xinjiang
W68	5,331,615,600	86.60%	97.30%	149.2	yellow	wild	Beijing
W55	5,559,805,200	92.10%	88.70%	147.8	yellow	wild	Shandong
HN213	9,280,081,500	86.00%	88.60%	146.1	yellow	wild	-
W69	4,878,531,000	91.90%	88.70%	145.7	yellow	wild	Heilongjiang
HB19101	4,991,530,200	91.60%	88.60%	145.4	yellow	wild	Hebei
W62	5,370,762,900	88.90%	88.50%	144.7	yellow	wild	Hebei
FGY171E	5,881,290,300	85.10%	88.70%	144.5	yellow	wild	Sichuan
CD913	4,840,125,600	83.30%	88.70%	144.3	yellow	wild	-
XD91S1	5,379,901,800	83.60%	90.40%	144.3	yellow	wild	Sichuan
W50	4,936,523,400	90.90%	88.70%	143.7	yellow	wild	Liaoning
XF91A	5,950,436,400	86.30%	91.30%	143.5	yellow	wild	Xinjiang
XB93	5,463,701,400	89.60%	88.70%	141.9	yellow	wild	Xinjiang
W49	4,947,195,300	90.90%	91.20%	141.7	yellow	wild	Liaoning
W54	4,989,796,500	92.00%	88.70%	140.7	yellow	wild	Shandong
W48	5,479,733,400	92.20%	89.10%	139	yellow	wild	Sichuan
W61	5,227,953,300	92.60%	87.80%	137.2	yellow	wild	Hebei
SY91A	5,682,915,300	85.50%	92.10%	130.6	yellow	wild	Liaoning
W45	4,996,704,900	89.70%	91.00%	126.9	yellow	wild	Sichuan
7AA	6,039,291,000	87.50%	90.20%	126.3	yellow	wild	Australia
W59	4,987,322,100	92.00%	88.90%	125.7	yellow	wild	Hebei
HL1703	6,084,036,300	88.60%	89.40%	125.6	yellow	wild	Sichuan
W43	4,937,946,000	82.90%	92.80%	125.4	yellow	wild	Sichuan
W60	4,909,563,000	88.30%	92.10%	123.3	yellow	wild	Hebei
FJL1503	5,474,032,200	86.70%	91.20%	122.1	yellow	wild	Jilin
HB1925	4,921,756,800	89.50%	89.80%	122.1	yellow	wild	Hebei
TC92S2	5,484,576,000	88.40%	92.10%	122.1	yellow	wild	Sichuan
BJ2	5,834,235,600	87.60%	90.50%	121.4	yellow	wild	Beijing
HNY6	6,984,377,400	88.90%	92.00%	121.2	yellow	wild	-
W42	5,374,737,000	87.50%	90.20%	119.4	yellow	wild	Sichuan
HB1924	5,373,926,100	88.40%	91.90%	117.6	yellow	wild	Hebei
HB1926	5,279,833,800	87.90%	90.90%	115.2	yellow	wild	Hebei
HB110	4,965,000,300	88.20%	92.40%	112	yellow	wild	Hebei
HB1931	5,207,298,000	89.00%	90.40%	111.3	yellow	wild	Hebei
HB19111	5,721,979,500	88.80%	92.80%	110.3	yellow	wild	Hebei
W47	5,151,517,800	89.30%	91.60%	109.1	yellow	wild	Sichuan
FCD176A1	5,711,787,300	86.30%	90.90%	108.9	yellow	wild	Chengdu
FSD175	5,342,841,600	88.20%	92.60%	108.7	yellow	wild	Chengdu
HB197	5,176,382,400	87.80%	90.30%	108.4	yellow	wild	Hebei
CD912_526	6,326,020,800	86.30%	89.90%	108	yellow	wild	Chengdu
BJ932	5,390,764,200	81.30%	91.40%	106.5	yellow	wild	-
BJ91A	5,930,747,700	83.80%	91.30%	104.6	yellow	wild	-
CD914_527	5,281,848,600	64.70%	90.90%	80.8	yellow	wild	Chengdu
FSD172C	5,748,301,500	63.90%	91.60%	79.9	yellow	wild	Chengdu
BJ945C	4,855,080,900	41.70%	91.20%	57.4	yellow	wild	Beijing
BJ91	5,108,668,500	37.10%	91.90%	50.1	yellow	wild	Beijing
W53	4,953,074,400	28.40%	88.30%	49.5	yellow	wild	Shandong
HB1961	4,988,508,600	32.80%	89.30%	43.9	yellow	wild	Hebei
B6512	13,623,971,700	92.30%	88.50%	321.6	white	cultivar	-
YY614	6,801,244,200	85.20%	88.70%	224.2	white	cultivar	-
C43	4,652,067,600	90.20%	88.70%	190.4	white	cultivar	Fujian
YR13	6,196,084,800	90.90%	97.80%	180.8	white	cultivar	-
WB210	7,274,784,900	91.60%	88.80%	180.7	white	cultivar	-
YD668	6,883,548,000	89.00%	91.70%	176.6	white	cultivar	-
XR2111	6,265,922,100	75.60%	91.50%	172.4	white	cultivar	-
C42	4,554,684,900	90.60%	88.70%	168.2	white	cultivar	Fujian
XR2101	5,978,366,100	90.00%	91.90%	163.9	white	cultivar	-
W536	6,755,451,000	85.60%	91.50%	163.2	white	cultivar	-
RD7	6,676,121,400	93.10%	88.50%	161.6	white	cultivar	Sichuan
BY9529	6,016,801,500	89.00%	88.70%	161.4	white	cultivar	-
XRH730	6,742,733,700	90.00%	88.60%	158.6	white	cultivar	-
WB228	5,533,026,600	89.60%	88.30%	157	white	cultivar	-
XR192	6,175,200,600	90.60%	89.20%	157	white	cultivar	Japan
W5ZJ	6,198,809,700	88.50%	91.20%	156.9	white	cultivar	-
YN92	5,416,198,800	90.70%	88.70%	155.9	yellow	cultivar	-
GR91-1	6,788,983,800	90.30%	88.30%	154.4	white	cultivar	-
GR91-1	6,788,983,800	90.30%	88.30%	154.4	white	cultivar	-
G013	5,320,581,300	90.80%	88.60%	153.9	white	cultivar	Japan
B32	5,648,609,700	91.90%	89.00%	153.7	white	cultivar	Sichuan
W527	6,783,499,800	85.80%	88.70%	149.3	white	cultivar	-
YRW1513	6,790,506,600	83.10%	97.80%	147.7	yellow	cultivar	-
9AA	6,002,955,600	91.40%	93.70%	147.5	white	cultivar	HongKong
WB141	5,889,080,100	84.10%	88.60%	147.3	white	cultivar	Japan
WC1501	6,640,416,000	90.30%	88.70%	147.1	white	cultivar	-
WB57	6,649,017,300	82.10%	88.70%	146.8	white	cultivar	-
Y56	5,334,398,400	85.70%	88.80%	146.3	white	cultivar	-
3AA	5,784,305,700	93.60%	97.40%	145.6	yellow	cultivar	Hebei
XR2011	6,867,214,800	88.80%	91.80%	145.1	white	cultivar	-
WS219	7,213,323,900	90.10%	91.30%	144.8	white	cultivar	-
YSR211	6,521,972,100	91.20%	93.70%	144.2	white	cultivar	-
RF92	5,552,944,800	90.60%	92.30%	143.8	white	cultivar	Japan
19AA	5,928,289,500	92.50%	96.20%	143.3	white	cultivar	Japan
5AA	5,330,144,700	92.60%	97.80%	142.1	yellow	cultivar	Hunan
GR91	5,641,761,300	90.90%	88.70%	141.8	white	cultivar	-
E338	6,093,056,400	88.20%	88.40%	141.6	white	cultivar	Japan
XGF2116	5,892,126,000	91.50%	88.70%	141	white	cultivar	-
RJ1	7,311,964,500	90.80%	88.80%	140.9	white	cultivar	-
BCT6	8,254,567,800	90.00%	88.60%	140.7	white	cultivar	-
13AA	5,966,978,100	91.60%	97.20%	139.7	yellow	cultivar	Fujian
17AA	6,035,661,600	90.40%	88.40%	138.8	white	cultivar	Shanghai
CH816	6,228,906,600	87.30%	89.90%	138.1	white	cultivar	-
G130Y	5,248,216,200	86.90%	88.60%	137.7	yellow	cultivar	-
18AA	6,025,245,900	88.90%	88.60%	137.1	white	cultivar	Japan
ACR412	6,455,527,800	90.40%	88.60%	136.5	white	cultivar	-
RC17	6,888,097,800	87.70%	88.70%	136.5	white	cultivar	-
A611	5,756,805,300	89.20%	88.50%	136	white	cultivar	Japan
15AA	6,039,788,100	88.30%	93.00%	135.8	white	cultivar	Hebei
BB9229	7,076,914,800	92.00%	88.70%	135.6	white	cultivar	-
16AA	6,014,332,500	87.60%	88.80%	135.2	white	cultivar	Fujian
531	6,054,606,900	91.70%	96.80%	134.5	white	cultivar	-
BCF1	6,116,104,800	81.60%	89.00%	134.4	white	cultivar	-
YW6514	6,854,542,500	87.60%	90.30%	134	white	cultivar	-
10AA	5,958,497,100	87.70%	88.50%	133	white	cultivar	Japan
2AA	5,055,837,600	85.90%	94.30%	132.7	yellow	cultivar	Guizhou
FBJ4	5,715,221,700	90.90%	98.00%	131.9	white	cultivar	Shanghai
RC7433	5,878,678,500	91.20%	88.70%	131.7	white	cultivar	-
A2_2	5,899,424,700	86.60%	90.30%	131.4	white	cultivar	Sichuan
CF2021	6,746,666,100	88.40%	92.20%	130.8	white	cultivar	-
6AA	5,466,440,700	84.20%	88.60%	130.3	white	cultivar	Liaoning
Y516	6,732,100,800	85.60%	88.70%	128.5	white	cultivar	-
BCF5	7,092,733,800	88.60%	88.70%	128.1	white	cultivar	-
YB66	5,790,196,200	90.00%	88.30%	128	white	cultivar	-
T7-3	6,441,932,700	87.00%	90.00%	127.9	white	cultivar	-
11AA	6,034,369,800	82.80%	88.50%	127.1	white	cultivar	Taiwan
F629	5,567,275,200	85.60%	92.20%	126	yellow	cultivar	-
JN1403	6,360,789,300	84.70%	89.70%	125.8	white	cultivar	-
HS213	6,842,381,400	88.50%	93.90%	125.5	white	cultivar	-
730FT	6,296,794,500	92.00%	97.80%	125.2	yellow	cultivar	Sichuan
JDG214	6,994,112,100	85.80%	91.10%	124.3	white	cultivar	-
JB1	6,854,586,000	83.60%	92.50%	122.4	white	cultivar	-
22AA	6,028,996,500	91.90%	97.20%	122.3	white	cultivar	Beijing
4AA	6,006,551,100	92.30%	95.40%	121.4	yellow	cultivar	Henan
CR51	5,990,160,600	79.00%	91.00%	121	white	cultivar	-
RY8	7,670,523,000	88.20%	92.10%	120.3	white	cultivar	-
8AA	5,137,149,900	85.50%	91.30%	118.7	white	cultivar	Australia
LFB11	6,313,579,200	85.50%	91.70%	118.3	white	cultivar	-
LRY2113	6,165,061,500	81.30%	89.10%	118.3	yellow	cultivar	-
1AA	5,212,569,900	77.10%	88.40%	116.4	yellow	cultivar	Yunnan
CJ631	6,686,406,300	85.20%	91.80%	116.4	yellow	cultivar	-
A45	6,532,073,700	88.60%	87.10%	115.3	white	cultivar	Japan
CJ6	5,651,249,100	80.40%	90.50%	114.5	yellow	cultivar	Sichuan
WY851	6,602,663,700	86.40%	91.20%	114.3	white	cultivar	-
RY2110	6,243,451,800	76.80%	90.50%	111.1	white	cultivar	-
DJ1601	6,199,958,700	91.00%	88.60%	108.1	white	cultivar	-
F093	6,686,192,400	88.00%	91.60%	108.1	white	cultivar	-
12AA	5,564,021,100	76.50%	88.20%	107.9	white	cultivar	Taiwan
RJ567	7,835,205,300	82.80%	90.10%	104.2	yellow	cultivar	-
YW6512	7,055,345,700	71.30%	90.40%	102	white	cultivar	-
E3	6,845,109,300	73.20%	91.80%	98.9	white	cultivar	-
SDY2114	6,203,431,800	74.50%	90.70%	97.5	yellow	cultivar	-
HL212	6,803,610,300	93.20%	93.50%	92.3	white	cultivar	-
RY8331	6,712,070,400	73.50%	91.00%	91.4	white	cultivar	-
LM216	6,020,644,200	89.70%	88.00%	91.2	white	cultivar	-
LFH3	5,185,681,800	91.60%	86.10%	90.4	yellow	cultivar	-
HL211	6,303,453,900	86.40%	86.60%	85.6	white	cultivar	-
GR91Y	6,230,154,600	89.90%	91.30%	79.6	yellow	cultivar	-
H13	6,061,021,500	89.50%	90.40%	71.4	white	cultivar	Japan
RW263	6,773,640,600	55.20%	91.00%	69.8	white	cultivar	-
CJY2115	6,064,386,900	44.00%	91.80%	53.7	yellow	cultivar	-

**Table 2:** The basic information of the MNP marker library.

Sample	Clean Reads	Average Coverage of MNP Markers	Number of MNP Marker Detected	Cap Color	Source
XF91	8217560	20,235	145	yellow	wild
BJ92	7584880	18,428	132	yellow	wild
HB171	7272776	17,543	144	yellow	wild
YAAS6018	12684464	31,165	133	yellow	wild
BJ954	7633094	19,057	150	yellow	wild
BJ4	8252300	17,824	134	yellow	wild
WS2147	8901998	22,015	166	yellow	wild
HB54	12034098	29,346	153	yellow	wild
JL211	8716782	21,508	141	yellow	wild
HD91	11488732	28,263	153	yellow	wild
CD911G	10396374	25,940	153	yellow	wild
HB1911	7681900	19,159	122	yellow	wild
SX816	6655314	16,590	138	white	factory cultivar Zhongxing
6B25	9585640	23,870	143	white	factory cultivar Zhongxing
CHQ19	7473658	18,585	137	white	factory cultivar Zhongxing
TS816	8376310	20,840	141	white	factory cultivar Zhongxing
HX218	6678532	16,748	120	white	factory cultivar Zhongxing
CHQ2	7716738	19,252	138	white	factory cultivar Zhongxing
CH213	9069074	22,606	141	white	factory cultivar Zhongxing
CH0708	10101620	25,129	131	white	factory cultivar Zhongxing
YH217	9477522	23,555	151	white	factory cultivar Youhong
NK1301	8298778	20,665	138	white	factory cultivar Xuerong
DJ1401	8074850	20,094	135	white	factory cultivar Xuerong
XR201	8891596	22,120	132	white	factory cultivar Xuerong
RYY2112	8744304	21,595	143	yellow	factory cultivar Ruyi
YP215	7452770	18,548	128	white	factory cultivar Ruyi
T8-4	11198626	27,941	134	white	factory cultivar Kangrui
HC211	7525108	18,780	131	white	factory cultivar Hualv
JDG221	7407104	18,453	139	white	factory cultivar Guangming
E3209	7651640	19,053	132	white	factory cultivar Gangrongtai
LMPQ6	8723392	21,702	137	white	factory cultivar Gangrongtai
G1A	7270186	18,124	132	white	factory cultivar Gangrongtai
E3202	8027632	20,002	137	white	factory cultivar Gangrongtai
E3818	8273004	20,610	134	white	factory cultivar Gangrongtai
E87	9291466	23,082	136	white	factory cultivar Gangrongtai
XGF216	8798058	21,892	135	white	factory cultivar
MY1201	8029920	19,940	141	white	cultivar (Taiwan)
HG91	9004562	21,389	141	yellow	cultivar (Korea)
T011	8770926	21,975	134	white	cultivar (Japan)
XQY2117	8060156	20,159	154	yellow	cultivar (Guangdong)
HR9820	8631350	20,746	143	white	cultivar
RY833	9599096	23,888	145	white	cultivar
5Y16	7134102	17,761	134	white	cultivar
YG99	9588270	23,787	150	yellow	cultivar
CJ10	9040974	22,227	156	yellow	cultivar
BCT1	7396960	18,446	139	white	cultivar
PY7812	8825106	22,022	147	white	cultivar
F103	7472462	18,523	163	white	cultivar
FV093	8856082	22,150	122	white	cultivar
3W4	9080266	22,622	147	white	cultivar
S7	8393386	20,712	153	yellow	cultivar
YG910	8663284	21,498	155	yellow	cultivar
YG95	7334222	18,219	159	yellow	cultivar
GCF36	10356488	25,876	146	white	cultivar
CJ57	8307228	20,691	125	white	cultivar
CJ58	8636466	21,516	137	white	cultivar
LPY2113	10584644	26,393	147	yellow	cultivar
W543	8411992	20,871	135	white	cultivar
YF33	8865860	21,905	145	white	cultivar
W119	7222580	16,310	122	white	cultivar
YW6518	7632486	18,976	128	white	cultivar
S6	8391306	20,817	151	yellow	cultivar
YD48	8491296	21,116	139	white	cultivar
5C27	12126742	30,149	130	white	cultivar
CJ3	7872246	19,513	148	yellow	cultivar
EG7	11316622	28,131	144	white	cultivar
S4	18481160	45,878	164	yellow	cultivar
S5	9782768	24,268	157	yellow	cultivar
S3	10382322	25,833	150	yellow	cultivar

## Data Availability

All the genomic sequence data sets are available in the NCBI Sequence Read Archive under accessions PRJNA905113.

## Supplementary Materials

The following supporting information can be downloaded at: https://www.mdpi.com/article/10.3390/jof9030330/s1, Table S1: the basic information of MNP marker library; Table S2: the GS values of pairwise comparison between 22 pedigrees and all cultivars; Table S3: the minimum genetic similarity values of 22 pedigrees.
